# C-GCN: A Flexible CSI Phase Feature Extraction Network for Error Suppression in Indoor Positioning

**DOI:** 10.3390/e23081004

**Published:** 2021-07-31

**Authors:** Wen Liu, Qianqian Cheng, Zhongliang Deng, Mingjie Jia

**Affiliations:** School of Electronic Engineering, Beijing University of Posts and Telecommunications, Beijing 100876, China; ambercheng@bupt.edu.cn (Q.C.); dengzhl@bupt.edu.cn (Z.D.); jiamingjie@bupt.edu.cn (M.J.)

**Keywords:** channel state information, phase, feature extraction, fingerprint localization

## Abstract

Channel state information (CSI) provides a fine-grained description of the signal propagation process, which has attracted extensive attention in the field of indoor positioning. However, considering the influence of environment and hardware, the phase of CSI is distorted in most cases. It is difficult to extract effective location features in multiple scenes only through the determined artificial experience model. Graph neural network has performed well in many fields in recent years, but there is still a lot of room to explore in the field of indoor positioning. In this paper, a phase feature extraction network based on multi-dimensional correlation is proposed, named Cooperation-Graph Convolution Network (C-GCN). The purpose of C-GCN is to extract new features of multiple correlation and to mine the relationship between antenna and subcarrier as much as possible. C-GCN is composed of convolution layer and graph convolution layer. In the graph convolution layer, C-GCN regards each subcarrier of each antenna as a node in the graph network, constructs the connection by the correlation between the antenna and the subcarrier, and aggregates the node vectors by graph convolution. In the convolution layer, there is a natural corresponding structure between data packets, C-GCN extracts the fluctuation with convolution in Euclidean space. C-GCN combines these two layers, and applies end-to-end supervised training to obtain effective features. Extensive experiments are conducted in typical indoor environments to verify the superior performance of C-GCN in restraining error tailing. The average positioning error of C-GCN is 1.29 m in comprehensive office and 1.71 m in garage. Combined with the amplitude feature, the average positioning error is 0.99 m in comprehensive office and 1.14 m in garage.

## 1. Introduction

With the popularity of mobile devices, location-based services (LBS) provide great convenience for smart cities, industrial production and people’s daily life. Because of the shelter of buildings and the complexity of indoor environment, Global Navigation Satellite System (GNSS) that widely used outdoors is unable to achieve high-precision positioning indoors. To solve this problem, assistant signal sources like Wi-Fi [1,2], Bluetooth [3,4], RFID [5] and ultra-wideband (UWB) [6,7] have been proposed to provide ample features. Localization methods including angle of arrival-based method (AOA) [8], time of arrival-based method (TOA) [9], and fingerprint- based method [10] are proposed to locate targets.

Commercial Wi-Fi devices have gradually opened up the physical layer information, making it possible to obtain channel state information (CSI), such as Intel Wi-Fi link 5300 NIC [11] and Atheros ar9580 chipset [12]. Compared with the received signal strength information (RSSI), CSI contains rich features from multiple antennas and subcarriers, providing a fine-grained description of the indoor environment.

CSI data consists of amplitude and phase. The amplitude feature is greatly affected by NLOS(Non Line of Sight) and noise, while the phase feature is distorted by sampling frequency offset, center frequency offset and packet delay et al. The existing research shows that the processed phase can effectively reduce the influence of occlusion [13], so this paper focuses on the phase feature extraction of CSI in the indoor environment.

In order to extract effective positioning feature from the original phase, Y. Zhuo et al. [14] discussed five factors that affect the phase accuracy: carrier frequency offset (CFO), sampling frequency offset (SFO), packet detection delay (PDD), PLL phase offset (PPO) and phase ambiguity (PA). M. Kotaru et al. [15] corrected the known linear error part by linear transformation. Y. Zhuo et al. [16] proposed a model to fit the nonlinear error. Xuyu Wang et al. proposed to extract phase features by self-encoder BiLoc [17] and convolutional neural network CiFi [13], and eliminate errors caused by PDD, SFO and CFO by calculating the phase difference between antennas.

The existing phase error correction algorithms are mainly based on the artificial experience nonlinear model and linear transformation, and the interaction between phases is mainly calculated by difference. Artificial experience model is only a partial reflection of the phase shift, which is often difficult to adapt to a variety of complex environments, and its adaptability in a variety of scenarios needs to be further verified.

With the upgrade of hardware computing ability, deep learning has been widely used in CSI based indoor positioning. In 2016, Wang et al. [18] proposed DeepFi system, which extracts features based on CSI amplitude by self-encoder, and takes the weights of the neural network as new fingerprint. Then position estimation is carried out based on probability algorithm. CFI [19] extracted feature of calibrated phase by self-encoder. The location errors in open and complex environments are 1.08 m and 2.01 m, respectively. In 2017, CiFi [13] calculated AOA by CSI phase difference, and assembled AOA into image. These images are sent to convolutional neural network for position estimation, and shows better performance than DeepFi and CFI. In 2019, Hsieh et al. [20] proposed one-dimensional CNN network. Based on the idea of classification, multiple one-dimensional convolution layers were used to deal with CSI amplitude and RSS respectively, and the results show that one-dimensional CNN can greatly reduce the network parameters while maintaining accuracy. BiLoc [17] improved the amplitude and AOA image respectively by self-encoder, and applied probability algorithm to match. Compared with the previous DeepFi, CFI and CiFi, which use single information, BiLoc uses both amplitude and phase, effectively improving the positioning accuracy. ResLoc [21] extracted the AOA and amplitude more accurately by the two-channel residual sharing learning mechanism.

Although deep learning has achieved great success in Euclidean data, more and more applications need to handle data from non-Euclidean space and carry out effective analysis. For example, building a recommendation system based on interpersonal relationship [19,22,23], material analysis based on molecular structure [24,25], and impact analysis based on Citation relationship [26,27]. There are unordered nodes with variable size in the non-Euclidean space, and the neighbors of each node is different, which is usually named graph data. Some important operations (such as convolution) are no longer directly applicable to the graph structure. In addition, a core assumption of existing machine learning algorithms is that instances are independent of each other. However, for graph data, each instance (node) is connected to each other through a complex connection. The complexity of graph data brings challenges to existing machine learning algorithms.

In 2013, Bruna [28] proposed a GCN variant based on spectral theory for the first time. Since then, there have been many improved spectral based convolution networks [26,29,30,31]. However, every time the spectral method is updated, it needs to deal with the whole graph, which is difficult to parallel or expand. So, the convolution network based on space has been widely concerned in recent years. Space-based convolution network convolutes the information of adjacent nodes directly into the graph domain, which improves the efficiency of operation.

In this paper, a phase feature extraction network based on multi-dimensional correlation is proposed, named Cooperation-Graph Convolution Network (C-GCN). C-GCN aims to extract new features with multiple correlations, excavate the relationship between antenna and subcarrier, then integrate the fluctuation of a single location into the database. Based on the time-space structure proposed by Chen Chen et al. [11]. There are two types of correlation in C-GCN: (1) correlation between antennas and subcarrier correlation in non-Euclidean space, (2) correlation between adjacent data packets in Euclidean space. A detailed analysis of these two correlations will be included in the design challenges in Section 3. C-GCN is mainly composed of convolution layer and graph convolution layer. In the graph convolution layer, C-GCN regards each subcarrier of each antenna as a node in the graph network, constructs the connection by the correlation between the antenna and the subcarrier, and aggregates the node vectors by graph convolution. In the convolution layer, there is a natural corresponding structure between data packets, C-GCN extracts the fluctuation with convolution in Euclidean space. C-GCN combines these two layers, and applies end-to-end supervised training to obtain effective location features.

The main contributions of this paper are summarized as follows [16]:

1 To the best of our knowledge, this is the first work to apply the graph convolution in CSI based indoor positioning. We elaborate on how to connect the data with the correlation between phases, and optimize the parameters with the constraints of the loss function to obtain new features that are effective for position estimation. This paper presents the feasibility of position estimation based on the irregular correlation in CSI.
2 Affected by random noise, the fingerprint fluctuation of a single location point is extracted from the data packet with natural corresponding data structure by Euclidean space convolution. This kind of time correlation only exists between nodes with the same subcarrier sequence number, which effectively reduces the impact of random noise during position estimation.
3 Extensive experiments are conducted in typical indoor environments to verify the superior performance of C-GCN in restraining error tailing. Besides, we also introduce amplitude features to further verify the feasibility of bi-modal positioning based on the new feature generated by C-GCN.

In the reminder of this paper, we provide the background of CSI in Section 2. Then the structure of C-GCN is presented in Section 3. The methodology is described in Section 4. Evaluation is implemented in Section 5. Section 6 summarizes this paper.

## 2. Preliminary

In Wi-Fi network, OFDM (Orthogonal Frequency Division Multiplexing) system divides communication channels into orthogonal subchannels with different frequencies. Channel state information (abbreviated as CSI) reflects the characteristics of communication link between transmitter and receiver. The data packet received by the receiver includes time stamp, RSSI, antenna number, noise, CSI et al. The transmission process in frequency domain can be expressed as:

(1) Y=HX+N

where *Y* is the received signal, *X* is the transmitted signal, *H* denotes the complex matrix of channel state information, *N* represents the additive Gaussian white noise, so CSI can be expressed as:

(2) $$\hat{H}=\frac{Y}{X}$$

where $\hat{H}$ represents the channel frequency response (CFR) of each subchannel.

The receiver correlates the received signal with the reference signal and samples at time zero to calculate the CSI. Assume that the reference complex sinusoidal signal of the frequency f transmitted by the q-th antenna is $e^{j2\pi ft}$, and the signal obtained through the wireless channel $h_{q}$ is $h_{q}e^{j2\pi ft}$. The corresponding CSI is calculated as follows:

(3) $${\hat{h}}_{q}=\frac{1}{T}\int_{0}^{T}h_{q}e^{j2\pi ft}e^{-j2\pi ft+jv}dt=h_{q}e^{jv}$$

where *T* is the transmission time and *v* is the relative phase between the receiver and the transmitter.

In the time domain, channel frequency response is expressed as channel impulse response (CIR). Its mathematical expression is as follows:

(4) $$h\left(\tau \right)=\sum_{i=1}^{N}am_{i}e^{-j\theta_{i}}\sigma \left(\tau -\tau_{i}\right)$$

$am_{i}$, $\theta_{i}$, $\tau_{i}$ represent the amplitude, phase and delay of the *i*-th signal propagation path respectively. *N* is the number of multipath, σ(τ) is the pulse function, and CFR and CIR are mutually Fourier transforms.

## 3. System Design

### 3.1. Design Challenges

The phase of CSI is mainly affected by carrier frequency offset, sampling frequency offset, packet detection delay, PLL phase offset and phase ambiguity. Linear factors are mainly eliminated by linear transformation, and nonlinear factors are generally fitted by artificial experience model. However, considering the complexity of obstacles and the serious impact of multipath in the indoor environment, the fixed artificial experience model is not good at adapting to the changing indoor scene. For the phase shift caused by unknown factors, there is no mainstream solution. To extract effective features from complex and changeable phase data is mainly faced with the following challenges:

First, in the calculation of CSI, a higher packet transmission rate is needed, so the receiver will get a large number of packets in a short time. Multiple time-dependent packets obtained at any reference point reflect the fluctuation of the signal. Traditional fingerprint database mainly stores reference fingerprint in a static way, which is easy to be affected by random noise. When matching online, it will bring uncontrollable random fluctuations. So how to apply temporal correlation to fingerprint database of CSI and improve the stability of location is still a problem to be solved.

**Figure 1:**
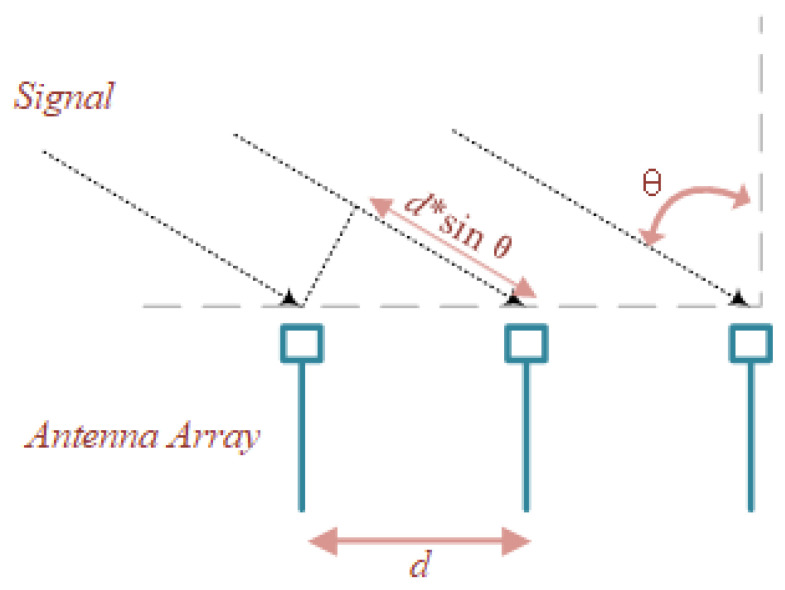
Uniform linear array model.

Second, each packet contains multiple subcarrier data from multiple antennas. Spotfi [32] system proposed the Phase relationship between antennas. In Figure 1, uniform linear array composed of 3 antennas, For AoA of θ, the target’s signal travels an additional distance of d∗sinθ to the second antenna in the array compared to the first antenna. There is a fixed distance between adjacent antennas, and the signal under the same path will introduce −2π∗d∗sin(θ)∗f/c phase difference, which can illustrate the correlation between antennas In addition, affected by multi-path, the delayed signal will have an offset in the frequency domain, so it will have a greater impact on adjacent signals. This impact is often not negligible, and it brings the uniqueness of the channel, which is often related to the ap location. So it’s necessary to explore this correlation introduced by multi-path. But the stability of the correlation is affected by the environment, which is Challenging to fit it with a fixed artificial experience model.

Three kinds of correlation including time correlation, antenna correlation and subcarrier correlation are discussed, which are also affected by random noise. How to combine them effectively is the key point of designing an algorithm to extract effective location features from complex phase.

### 3.2. System Architecture

**Figure 2:**
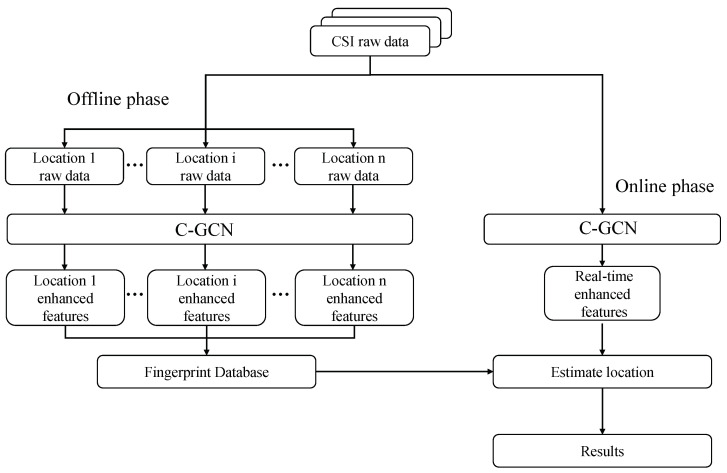
Structure of CSI phase feature extration system.

In this paper, a phase feature extraction network based on multi-dimensional correlation is proposed, named Cooperation-Graph Convolution Network (C-GCN). As a feature extraction module, C-GCN is applied to both offline and online to extract effective phase features. The structure of the positioning system is shown in Figure 2.

The offline part divides the original data into several groups according to the data labels and sends them to C-GCN to obtain the corresponding enhanced features as the fingerprint database. The online part collects real-time CSI phase data and computes new features in the same way. The last step is to match the real-time data with the fingerprint database and calculate the location of the target.

**Figure 3:**
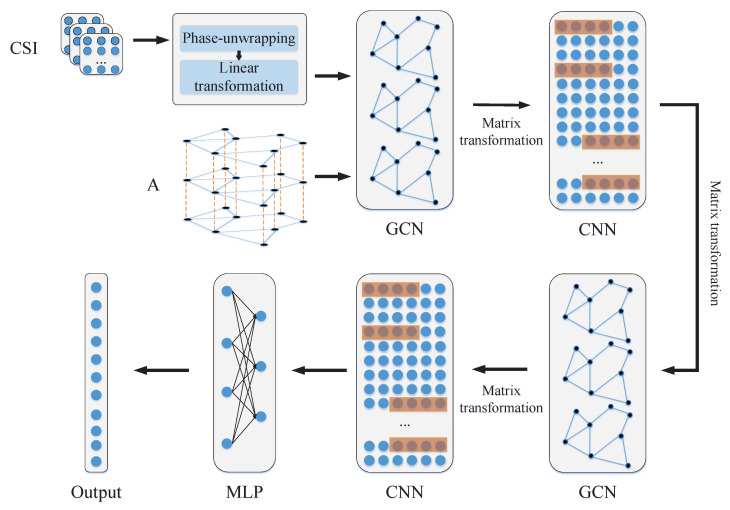
Structure of C-GCN.

The structure of C-GCN is shown in Figure 3. The input includes the original phase data and the adjacency matrix *A*. Adjacency matrix is the representation of the connection between data. The preprocessing of the original phase includes two steps: phase unwrapping and linear transformation. Phase unwrapping is used to remove the period introduced by arctangent function, and linear transformation is applied to remove the known linear factors.

C-GCN is mainly composed of convolution layer and graph convolution layer. The convolution layer is used to capture the fluctuation of multiple data packets of a single location over time. There is a natural corresponding structure between multiple data packets, which belongs to Euclidean spatial correlation. C-GCN adds the time correlation to the weight optimization through the convolution layer. The graph convolution layer is applied to capture the correlation between antenna and subcarrier in a single packet, which is more flexible and exists both in adjacent antennas (or subcarriers) and one to many antennas (or subcarriers). In the graph convolution layer, each subcarrier of each antenna in a single packet is viewed as a node in the graph network, and the edge connection of the graph is established by antenna correlation and subcarrier correlation to simulate the information transmission between nodes. The specific connection strategies and mathematical expressions are described in 4.1.1. Inspired by the inception module in GoogLeNet [19], we use a GCN and a CNN as a block, and we call this block twice in our network to achieve the best results. The end-to-end supervised learning is applied to calculate the optimal parameters and iteratively minimize the loss function.

## 4. Methodolgy

The details of C-GCN is introduced in this section, which consists of two parts: (1) graph convolution layer in non-Euclidean space and (2) convolution layer in Euclidean space.

### 4.1. Graph Convolution Layer in Non-Euclidean Space

The graph convolution layer extends the convolution from the traditional Euclidean space to the non-Euclidean space. The key point of graph convolution layer is to aggregate the feature $X_{i}$ of node $v_{i}$ and the feature $X_{j}$ of its neighbor, and generate a new representation of node *v*’s feature by learning function *f*, where $j\in N(v_{i})$. The process of graph convolution consists of three steps: (1) aggregate the neighbor node of vector *v*, (2) aggregate neighbor nodes with *v*, and (3) aggregate all nodes. The mathematical expressions of the above three parts are:

(5) $$a_{v}^{\left(k\right)}=AGGREGATE^{\left(k\right)}\left(\left\{z_{u}^{\left(k-1\right)}:u\in N\left(v\right)\right\}\right)$$

(6) $$z_{v}^{\left(k\right)}=COMBINE^{\left(k\right)}\left(z_{v}^{\left(k-1\right)},a_{v}^{\left(k\right)}\right)$$

(7) $$z_{G}=READOUT\left(\left\{z_{v}^{\left(k\right)}|v\in G\right\}\right)$$

where *u* is the neighbor node of *v*, $z_{v}^{\left(k-1\right)}$ represents the k−1-order aggregation of node *u*, $a_{v}^{\left(k\right)}$ represents the *k*-order aggregation result, $z_{v}^{\left(k\right)}$ represents the aggregation result with the node itself, $z_{G}$ represents the aggregation result of all nodes, which is used for graph vector representation.

#### 4.1.1. Graph Convolution Strategy

Correlation in C-GCN can be divided into two types: correlation in single data package and correlation in multiple data packages. This section focuses on the relationships in a single packet. The mathematical expression of a single packet is shown in Equation (8), where *n* is the number of antennas and *m* is the number of subcarriers. There are two kinds of relations in a single packet: the correlation between antennas $(h_{\left(i,j\right)},h_{\left(i,j^{\prime }\right)})$ and the correlation between subcarriers $(h_{\left(i,j\right)},h_{\left(i^{\prime },j\right)})$.

(8) $$CSI=\left[\begin{array}{cccc} h_{1,1} & h_{1,2} & \cdots & h_{1,n}\\ h_{2,1} & h_{2,2} & \cdots & h_{2,n}\\ \vdots & \vdots & \ddots & \vdots \\ h_{m,1} & h_{m,2} & \cdots & h_{m,n} \end{array}\right]$$

The correlation is expressed in the form of number pairs, and then converted to the adjacency matrix for calculation. The representation of correlation in pairs of numbers brings great flexibility to the customization of graph network. It supports both the representation of near neighbor relationship and the “far neighbor” after multi-level jump. Taking the adjacent node relationship as an example, *m*-subcarriers establish m−1 number pairs with neighbors, and *n* antennas establish n−1 number pairs with neighbors. These number pairs are transformed into adjacency matrix representation, in which the position with correlation is set as 1, and the other is 0. Based on adjacency matrix, undirected graph is constructed. The node set in the graph can be expressed as:

(9) $$V=\{V_{i}|i=1,2,\ldots ,N\}$$

where N=m∗n, it has better adaptability under different bandwidth and center frequency. The connection between nodes consist of two subsets, which can be expressed as:

(10) $$ES=\{V_{mi}V_{mj}|\left(i,j\right)\in N\}$$

(11) $$EF=\{V_{mi}V_{(m+1)i}\}$$

ES represents inter antenna correlation, which exists between corresponding subcarriers of different antennas. EF represents the correlation between subcarriers, which exists between different subcarriers of a single antenna.

#### 4.1.2. Aggregate Feature Vector of Nodes

The aggregation of node *v*’s neighbor nodes and the aggregation of neighbor nodes with *v* are realized by the following formula:

(12) $$Z^{\left(l+1\right)}=\sigma \left(D^{-\frac{1}{2}}AD^{-\frac{1}{2}}Z^{\left(l\right)}W^{\left(l\right)}\right)$$

where *A* is the adjacency matrix, *D* is the Laplace normalization matrix, $Z^{\left(l\right)}$ is the output of the previous layer, and $W^{\left(l\right)}$ represents weight matrix. The adjacency matrix *A* provides the connection relationship between adjacency nodes, and adds the self-connection by adding with the unit matrix *I*.

#### 4.1.3. Aggregate All Nodes into Graph

In the graph convolution layer, subcarrier data from each antenna in a single packet is regarded as nodes in the network, and the edge connection is established by the correlation between antenna and subcarrier. It is necessary to estimate the location based on all data in a package, so C-GCN is a graph level network, graph fusion is applied by pooling, as shown in the following:

(13) $$z_{v}^{\left(k\right)}=\mathrm{ReLU}\left(W*Mean\left\{z_{u}^{\left(k-1\right)},\forall u\in N\left(v\right)\cup \left\{v\right\}\right\}\right)$$

### 4.2. Convolution Layer in Euclidean Space

The receiver acquires a large number of CSI packets in a short time, which constitutes a three-dimensional data space. Graph convolution layer is used to calculate the correlation in a single data package in 2D space, convolution layer focuses on the temporal correlation among multiple data packets.

**Figure 4:**
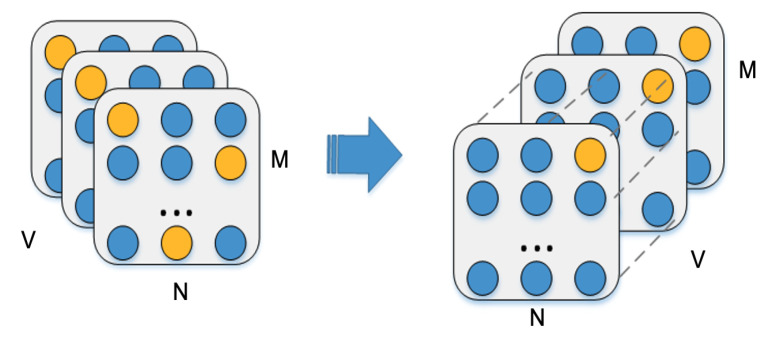
Graph connection in single packet and natural corresponding connection between multiple packets.

The data structure in the three-dimensional space is shown in the Figure 4. When the number of antennas and subcarriers is fixed, each two-dimensional slice has a natural corresponding relationship. Traditional static fingerprint does not record the fluctuation of data packets with time, so it is susceptible to burst noise in real-time calculation. This section introduces a dynamic fingerprint extraction layer based on CNN to improve the stability.

The input of convolution layer is the output of the previous graph convolution layer. Each packet contains a fixed amount of data, and there is a structure corresponding relationship between different packets. For this typical Euclidean spatial relationship, the information of adjacent packets can be aggregated by CNN. The connection between packets only exists between the corresponding subcarriers, which is used to simulate the association between multiple subcarriers at different times. As shown in Figure 4, the output matrix H(M,N,V) of the previous layer is converted to H(M,V,N), and then convolution is performed along the time axis.

### 4.3. Network Optimization

Over fitting will weaken the generalization ability of neural network. When the model is far beyond the task complexity, the performance of the model is superior in the existing training set, but poor in the new data set. This part optimizes the C-GCN from three aspects: dataset, network structure and training time.

In terms of dataset, the input of C-GCN includes adjacency matrix and preprocessed phase. Without special preprocessing of noise, it will propagate with the network layer. When the loss function is optimized, the interference term caused by noise is punished at the same time, so as to suppress the weight in the back propagation.

In terms of network structure, dropout is used to increase the randomness of the original structure by randomly invalid partial neuron nodes, so that the results are determined by multiple random structures.

In terms of training time, over fitting is avoided through early stopping. After each training, the accuracy of the verification dataset is recorded and compared with the optimal accuracy. If the continuous *t* times is lower than the optimal accuracy, the training is stopped. *T* is the upper limit, which is 20 in C-GCN.

In this section, C-GCN is optimized by the above three ways to increase the generalization ability.

## 5. Evaluation

### 5.1. Experiment Methodology

The positioning stability and accuracy of C-GCN are tested in two typical indoor scenarios: comprehensive office and garage. Besides, the feasibility of building a bi-modal positioning system by combining the C-GCN with amplitude is tested. The amplitude feature is the enhanced amplitude proposed in [33]. Both transmitter and receiver are mobile terminals equipped with Intel 5300 NIC. Each terminal is equipped with Ubuntu16.04. In the receiver, fine-grained CSI data is parsed by modifying the driver. The received packets include time stamp, RSSI, number of antennas, noise and CSI et al.

The Data set size is 226,000, of which 70% is the training set, 20% is the test set, and 10% is the validation set. Table 1 shows the experimental parameters. The specific parameters are set as follows:batch-size = 10, patience = 20, Learning-rate = 0.0005, Level-jump = 1. The activation function is sigmoid and the loss function is cross-entropy loss. we use the phase unwinding to preprocess the input In the training phase, we use the Glorot initializer to initialize the network. The Gradient change learning algorithm is Adam. And also we use the early stopping strategy, the initial best loss = $1\times 10^{9}$, patience = 20.

#### 5.1.1. Comprehensive Office

**Figure 5:**
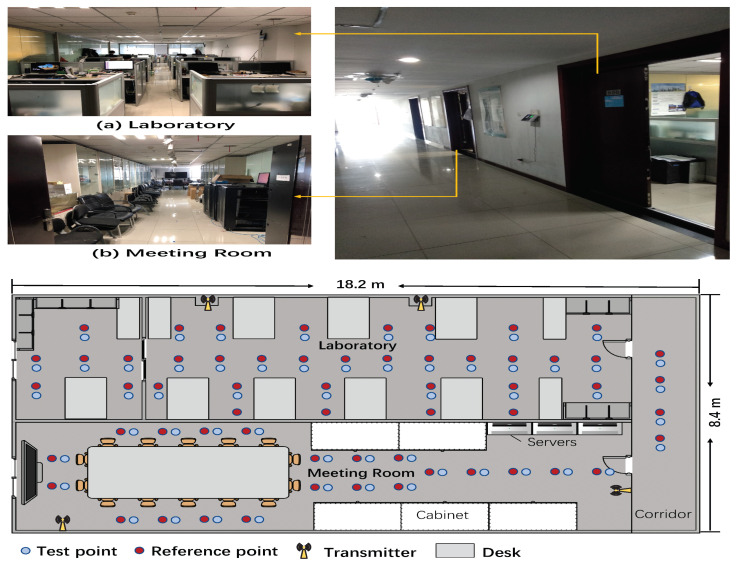
Layout of comprehensive office.

As shown in Figure 5, this is a comprehensive office environment including laboratory, conference room and corridor. The total area is 152.9 m^2^, the laboratory is 16.4 × 4.4 m^2^; the conference room is 16.4 × 4 m^2^; and the corridor is 8.4 × 1.8 m^2^. There are many tables and computers in the environment. The laboratory and conference room are separated by a glass wall. The layouts of this comprehensive environment are shown in Figure 4. There are 59 reference points, 55 test points and 4 transmitters. The distance between two adjacent reference points is 1.2 m. Our experiments were conducted outside of working hours, but there were still few people move around.

#### 5.1.2. Garage

**Figure 6:**
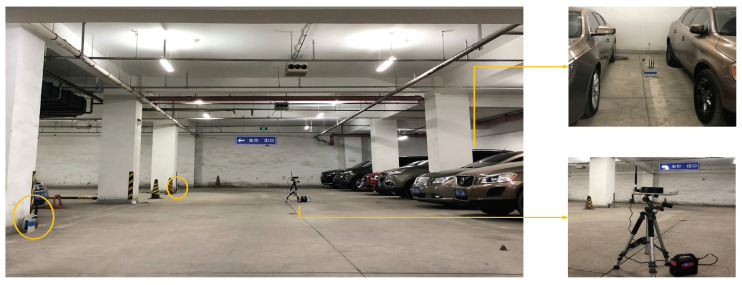

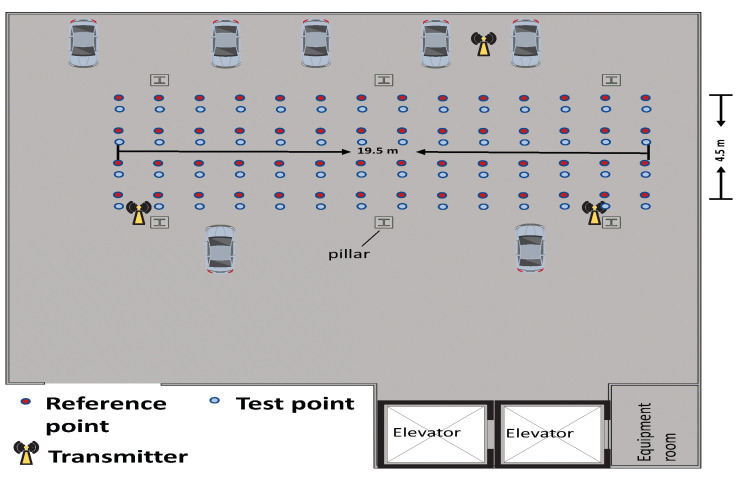
Layout of garage.

As shown in Figure 6, the second environment is an underground garage. This is a relatively special indoor environment that contains the movement of large metal objects (cars). The layouts are shown in Figure 5, the garage is 87.8 m^2^ with a total of 56 reference points, 56 test points and 3 emitters. The distance between two adjacent reference points is 1.5 m.

#### 5.1.3. Benchmarks

For comparison, two systems are implemented: CiFi [13] and BiLoc [17]. CiFi applies CNN to AOA image calculated by CSI’s phase feature, and BiLoc extracts bi-modal features based on self-encoder. For fair comparison, all three schemes use the same dataset.

Firstly, the single-modal features of C-GCN and CiFi are compared to verify the performance of C-GCN based on phase feature extraction. Then, the enhanced amplitude feature from [33] is introduced to construct our bi-modal positioning system MAP-GCN. Compared with BiLoc, the new features of C-GCN are verified to be feasible for bi-modal positioning.

### 5.2. Positioning Accuracy

#### 5.2.1. Single-Modal Estimation

**Figure 7:**
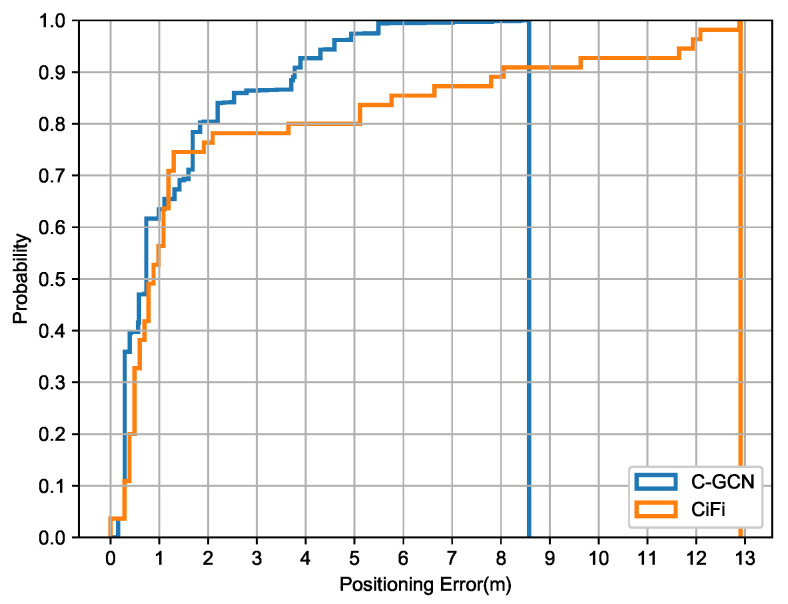
The cumulative positioning error of C-GCN and CiFi in comprehensive office.

The cumulative positioning error of C-GCN and CiFi in the comprehensive office is shown in Figure 7. In the range of 0–2 m, both of them perform good positioning ability, and C-GCN is slightly better. Generally speaking, CiFi performs well at the beginning, but the trend is not stable, which brings great uncertainty to the positioning of practical application. C-GCN has an obvious inhibition effect on large-scale deviation, which balances the positioning accuracy in the range of 0–2 m and effectively suppresses the problem of error tailing.When the probability reaches 1.0, the positioning error of C-GCN is 8.72 m, while the error of CIFI is 12.93 m.

**Figure 8:**
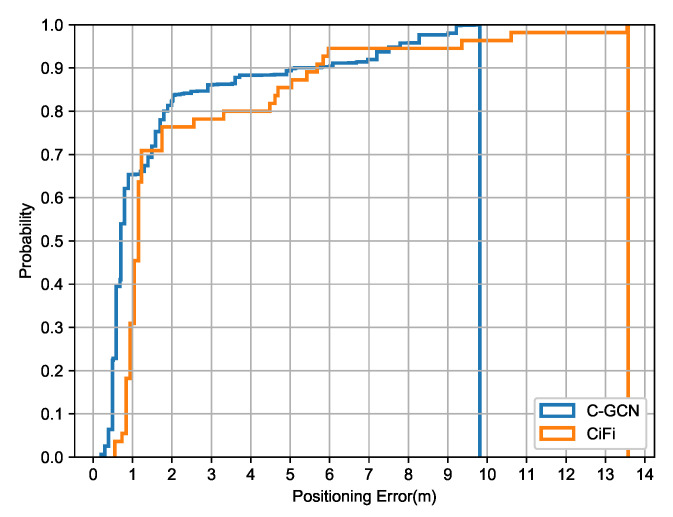
The cumulative positioning error of C-GCN and CiFi in garage.

Figure 8 shows the cumulative positioning error of C-GCN and CiFi in the garage. As can be seen from the figure, two curves intersect at 1–2 m and 5–8 m, When the probability reaches 1.0, the positioning error of C-GCN is 9.89 m, while the error of CIFI is 13.56 m. C-GCN but the performance of C-GCN is more stable and the positioning error is smaller. In the garage environment, the maximum positioning error has increased, but C-GCN still shows a good error suppression, effectively reducing the error fluctuation range.

#### 5.2.2. Bi-Modal Estimation

**Figure 9:**
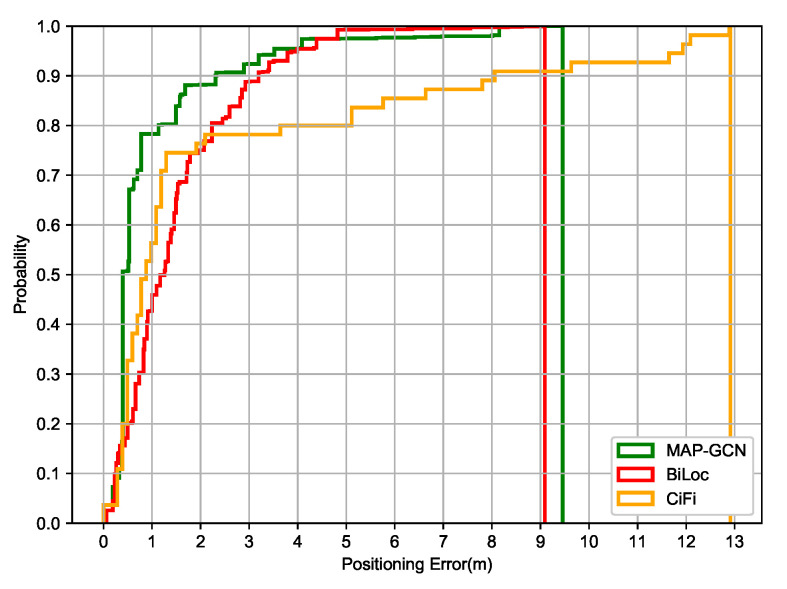
The cumulative positioning error of MAP-GCN, BiLoc and CiFi in comprehensive office.

Figure 9 shows the cumulative positioning error of C-GCN, BiLoc and CiFi in the comprehensive office. In the range of 0–2 m, both MAP-GCN and CiFi show good positioning performance. Combined with Figure 6, the early fluctuation of C-GCN is compensated. BiLoc also has a good inhibition effect on error tailing, but its positioning performance is relatively weak in the range of 0–2 m. At the same time, there is still a problem of error tailing in CiFi.When the probability reaches 1.0, the positioning error of MAP-GCN is 9.03 m and BiLoc is 9.35 m, while CIFI is 12.93 m.

**Figure 10:**
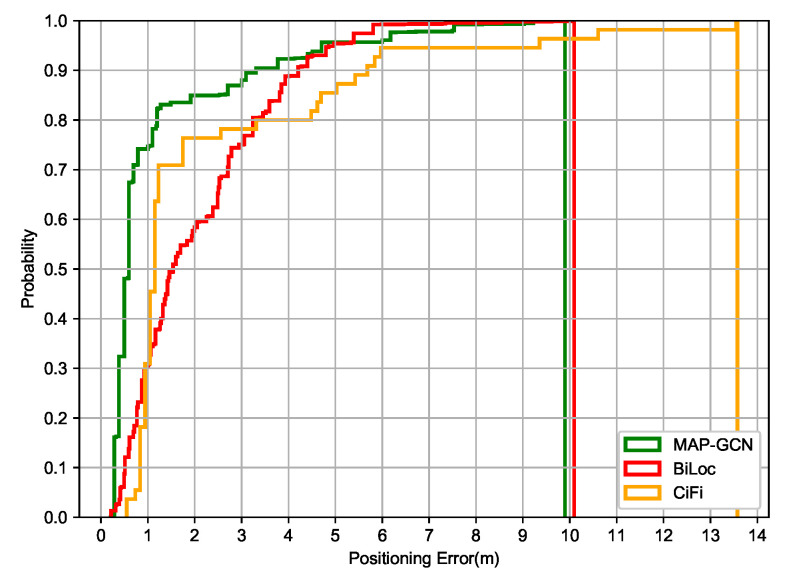
The cumulative positioning error of MAP-GCN, BiLoc and CiFi in garage.

Figure 10 shows the cumulative positioning error of MAP-GCN, BiLoc and CiFi in the garage, which is slightly worse than that in the comprehensive office environment. In the range of 0–2 m, the performance of MAP-GCN and CiFi is better than that of BiLoc, but the problem of error tailing still exists in CiFi. On the whole, both MAP-GCN and BiLoc can handle the error tailing problem, but in the range of 0–2 m, the positioning accuracy of BiLoc is relatively weak.When the probability reaches 1.0, the positioning error of MAP-GCN is 9.99 m and BiLoc is 10.02 m, while CIFI is 13.56 m.

### 5.3. Positioning Stability

This part will evaluate the positioning stability from mean error and standard deviation.

#### 5.3.1. Single-Modal Estimation

According to Table 2, compared with CiFi, the mean error of C-GCN is reduced by 46% and the standard deviation of positioning is reduced by 59%. Combined with the cumulative positioning error, it can be seen that the main reason for improving the mean error of C-GCN is to restrain the error tailing.

Table 3 shows detailed error comparison of C-GCN and CiFi in the garage environment. Compared with CiFi, the mean error of C-GCN is reduced by 23.6%, and the standard deviation is reduced by 16%. C-GCN effectively improves the positioning stability, ensures the positioning accuracy within the range of 0–2 m, and shows superior performance in terms of accuracy.

#### 5.3.2. Bi-Modal Estimation

This part evaluates MAP-GCN, CiFi and BiLoc from mean error and standard deviation.

The testing results in comprehensive office are shown in Table 4. It can be seen that MAP-GCN has superior performance in positioning accuracy. BiLoc performs well in positioning stability, and the average accuracy and standard deviation of CiFi are relatively weak.

Table 5 shows the detailed error comparison of MAP-GCN, BiLoc and CiFi in the garage. It can be seen from Table 5 that the performance in garage environment is similar to that in the comprehensive office, the performance of CiFi is not ideal considering the error tailing problem.

## 6. Summary

The phase feature of CSI is greatly affected by sampling frequency offset, center frequency offset and nonlinear noise, phase distortion and feature extraction are difficult. In this paper, three kinds of correlation among phases are analyzed: time correlation introduced by adjacent packets, antenna correlation introduced by antenna physical location, and subcarrier correlation. Based on the above, a CSI phase feature extraction network C-GCN based on multi-dimensional correlation is proposed. The network consists of convolution layer in Euclidean space and graph convolution layer in non-Euclidean space. The Euclidean space convolution is used to process the packet with natural structure, and the dynamic fluctuation at single point is integrated into the fingerprint database; the non-Euclidean space graph convolution layer is used to process the irregular relationship between antennas and subcarrier, and fully mine the correlation between them. Finally, we combine these two spaces and use the end-to-end training method to calculate new features including three types of correlation. C-GCN ensures the accuracy and stability of localization, and verifies the feasibility of positioning based on irregular correlation in CSI.The average positioning error of C-GCN is 1.29 m in comprehensive office and 1.71 m 18 in garage. Combined with the amplitude feature, the average positioning error is 0.99 m in 19 comprehensive office and 1.14 m in garage.And the application of this algorithm in aerial scenes remains to be explored.

In this paper, the validity of C-GCN in extracting phase feature is verified. The phase of CSI can still be explored from physical layer, and the phase can be further modified from the source. Accurate source data will improve the performance of feature extraction. More scene data can enrich the experiment content.

## Figures and Tables

**Table 1:** Parameters in the experiment.

Parameter Name	Parameter Value
batch-size	10
patience	20
Learning-rate	0.0005
loss function	cross-entropy loss
initializer	Glorot initializer

**Table 2:** Mean error and standard deviation in comprehensive office.

	C-GCN	CiFi
Mean error (m)	1.29	2.42
Standard deviation (m)	1.42	3.47
Minimum error (m)	0.02	0
Q1 (m)	0.12	0.92
Q2 (m)	0.94	1.12
Q3 (m)	1.91	2.11
Maximum error (m)	8.72	12.93

**Table 3:** Mean error and standard deviation in garage.

	C-GCN	CiFi
Mean error (m)	1.71	2.28
Standard deviation (m)	2.21	2.64
Minimum error (m)	0.02	0
Q1 (m)	0.96	0.97
Q2 (m)	0.99	1.12
Q3 (m)	1.85	1.93
Maximum error (m)	9.89	13.56

**Table 4:** Mean error and standard deviation in garage.

	MAP-GCN	BiLoc	CiFi
Mean error (m)	0.99	1.5	2.42
Standard deviation (m)	1.51	1.22	3.47
Minimum error (m)	0.01	0	0.47
Q1 (m)	0.35	1.39	0.34
Q2 (m)	0.44	1.84	0.46
Q3 (m)	1.42	3.12	1.56
Maximum error (m)	9.99	10.02	13.56

**Table 5:** Mean error and standard deviation in comprehensive office.

	MAP-GCN	BiLoc	CiFi
Mean error (m)	1.14	2.07	2.28
Standard deviation (m)	1.69	1.51	2.64
Minimum error (m)	0	0	0
Q1 (m)	0.31	0.46	0.32
Q2 (m)	0.32	1.28	0.67
Q3 (m)	1.12	2.01	1.88
Maximum error (m)	9.03	9.35	12.93

## Data Availability

Not applicable.
